# Serotonin Syndrome Precipitated by the Use of Cocaine and Fentanyl

**DOI:** 10.7759/cureus.22805

**Published:** 2022-03-03

**Authors:** Arshan Khan, Abdelilah Lahmar, Haris Asif, Muhammad Haseeb, Kelash Rai

**Affiliations:** 1 Internal Medicine, Ascension St. John Hospital, Detroit, USA; 2 Medicine, Faculty of Medicine and Pharmacy of Oujda, Oujda, MAR; 3 Internal Medicine, Woodhull Medical Center, New York, USA; 4 Internal Medicine, Bahria International Hospital, Lahore, PAK; 5 Internal Medicine, Jinnah Hospital, Lahore, PAK; 6 Internal Medicine, Ascension Providence Rochester Hospital, Rochester, USA

**Keywords:** serotonin syndrome and cyproheptadine, serotonin syndrome prognosis, serotonin syndrome in drug users, serotonin syndrome and fentanyl patch, serotonin syndrome and autonomic instability, serotonin syndrome management, serotonin syndrome diagnosis, serotonin syndrome precipitated by the use of cocaine and fentanyl, serotonin syndrome

## Abstract

Serotonin syndrome (SS) is a condition that occurs following the administration of serotonergic drugs. The syndrome is classically associated with the simultaneous administration of two serotonergic agents. Herein, we present a unique case of SS precipitated by cocaine and fentanyl in conjunction with previously prescribed oxycodone/paracetamol, trazodone, and mirtazapine. The patient was brought to the hospital with chief complaints of altered mental status, abdominal pain, nausea, and vomiting. The patient used her friend's fentanyl patch, and her urine drug screen was positive for cocaine. She was intubated and admitted to the intensive care unit for a low Glasgow Coma Scale score of 6 and autonomic instability. All the inciting agents were stopped, supportive treatment was given, and the patient was sedated with benzodiazepines and propofol. She received cyproheptadine, and the patient was extubated and clinically improved over the next 24 hours. Cocaine and fentanyl are not classically associated with SS. We did not encounter any cases where SS was precipitated by the combined use of cocaine and fentanyl in patients taking psychotropic medications during our literature review. This case report underlines the association of SS with cocaine and fentanyl. SS should be suspected in patients using cocaine and fentanyl or any other substance of abuse along with psychotropic agents.

## Introduction

Serotonin syndrome (SS) corresponds to a set of more or less severe, potentially fatal symptoms associated with an excess of serotonin in the central and peripheral nervous system, occurring after using serotonergic agents. It can be caused by drug use, drug overdose, or drug interactions [1]. This condition is classically manifested by a triad of cognitive-behavioral and neuromuscular disorders and damage to the autonomic nervous system. It has a variable prognosis, ranging from mild to severe to fatal forms [1,2]. The diagnosis of SS is clinical. The medication history must be meticulously collected. The first step is to stop taking the medication in question. Most cases usually resolve within 48 to 72 hours of stopping the causative agent. In more severe cases, hospitalization may be required to provide intensive treatment [3]. Here, we present a rare case of SS following the use of a fentanyl patch and cocaine in a patient who was previously prescribed oxycodone/paracetamol, trazodone, and mirtazapine.

## Case presentation

A 37-year-old female with a past medical history of bipolar disorder was presented to the hospital by the Emergency Medical Services (EMS) with chief complaints of confusion, generalized abdominal pain, nausea, and vomiting. The last known well time was 9 pm the night before the presentation. The patient's fiancé found the patient on the floor at 5 am, and she had a witnessed seizure and an episode of urine incontinence. The patient's fiancé stated that she took a fentanyl patch from one of her friends, applied the patch, and then went to bed the night before the presentation. After reviewing the medication history, the patient was prescribed oxycodone/paracetamol, trazodone, and mirtazapine.

On EMS arrival, the patient's pupils were less than 2 mm. The patient was given 0.2 mg of naloxone, and after receiving the naloxone, the patient had a seizure. The patient was given 5 mg of intravenous midazolam for seizure and was brought to the emergency room.

In the emergency department, the patient was hypertensive with a blood pressure of 165/81 mmHg and tachycardic with a heart rate of 184 beats/minute. Her respiratory rate was 39 breaths/minute, and the temperature was 104°F. The patient was extremely agitated and was placed in a four-point restraint. On physical examination, the patient's pupils were dilated and nonreactive to light, with extreme total body rigidity, ocular clonus, and inducible and spontaneous myoclonus of the lower extremities with hyperreflexia (grade 4).

The laboratory results on admission were as follows: white blood cell (14,000 cells/mcL), sodium (132 mEq/L), chloride (96 mmol/L), bicarbonate (16 mmol/L), lactic acid (5.5 mmol/L), and alkaline phosphatase (129 IU/L). The urine drug screen was positive for cocaine metabolites, opiates, cannabinoids, and benzodiazepine. Electrocardiogram revealed sinus tachycardia and was otherwise unremarkable. CT of the head without contrast was unremarkable for any acute intracranial pathology.

The patient was given multiple doses of intravenous lorazepam because of agitation and autonomic instability (hyperthermia, tachypnea, tachycardia, diaphoresis, or mydriasis). Later on, the patient was intubated because of a Glasgow Coma Scale (GCS) score of 6. The patient was started on propofol for sedation and agitation. External cooling was initiated for hyperthermia with a target temperature of 98°F. The patient was diagnosed with SS clinically with the help of Hunter's criteria. The patient was given cyproheptadine 12 mg through a nasogastric (NG) tube. The poison control team was contacted, and they agreed with the diagnosis of SS and management. The patient was admitted to the medical intensive care unit (MICU) and was maintained on 4 mg cyproheptadine every four hours through an NG tube. The patient was extubated the next day, and over the next few days, the patient was safely discharged home with education about drug-drug interaction.

## Discussion

SS is a set of symptoms resulting from increased serotonergic activity. Indeed, this toxic syndrome can be triggered by the introduction or escalation of a serotonergic treatment or, most commonly, by the combination of multiple treatments with serotonergic effects [1]. Serotonergic agents include specific serotonin reuptake inhibitors (SSRIs), monoamine oxidase inhibitors (MAOIs), lithium, opiates, tricyclic antidepressants, and recreational drugs like cocaine amphetamines and derivatives such as ecstasy (MDMA, 3,4-methyl​enedioxy​methamphetamine) [4]. An excess of serotonin in the central nervous system is associated with SS. Different types of serotonergic receptors are likely to bind to serotonin. 5-HT 1A, 2A, and 3 receptors play a key role in the pathogenesis of SS. It is known that some drugs interfere with serotonin metabolism and increase the level of serotonin in the synaptic cleft, leading to receptor saturation [5].

In our case, the patient had applied a patch of fentanyl before going to sleep. Before her presentation to the emergency room, she was at her baseline, which raised suspicion of an association with SS. Fentanyl is a synthetic opioid commonly used to relieve moderate to severe chronic pain. This molecule binds to the mu receptor at the spinal, supraspinal, and peripheral levels, producing, in particular, an analgesic effect as well as a sedative effect. The common routes of administration are intravenous, transdermal, and transmucosal [6]. Fentanyl is widely utilized as an induction agent in anesthesia. It is also used in intensive care units and for procedural sedation with benzodiazepines. In a prospective observational study of 309 patients, 7.8% were diagnosed with SS, while most patients received two or more serotonergic drugs. The authors report that the diagnosis is not infrequent in intensive care and that the diagnosis may go unnoticed, particularly in the setting of increased use of serotonergic agents [7].

Furthermore, it is known that the incidence of SS is significantly higher in patients receiving fentanyl and a serotonergic agent [8]. However, there was a case report where the use of fentanyl was the sole causative agent in the absence of other drugs known to induce SS [8]. The amount of blood flow through the skin to which the patch is placed can also affect the rate at which the drug is absorbed. Any rise in skin temperature caused by fever, external application of heat, muscle activity, or local inflammation can gradually increase blood flow to the skin. Higher perfusion leads to increased systemic absorption and higher fentanyl serum concentrations [9]. It is also possible to inject fentanyl from a matrix patch. Cocaine inhibits serotonin uptake, and its combination with serotonergic drugs such as fentanyl may have a synergistic effect [2], as was the case in this patient. It is also worth noting that illicit fentanyl is occasionally mixed with other drugs, such as cocaine. DiSalvo et al. described a case series of nine patients who presented to the emergency department with opioid toxicity after inhaling illicitly manufactured fentanyl (IMF) from what they thought was cocaine [10].

The symptoms of SS are best described with a triad that includes mental status changes (confusion, delirium, convulsions, anxiety, and coma), neuromuscular changes (tremors, clonus, and hyperreflexia), and autonomic nervous system instability (tachycardia, tachypnea, hypertension, hypotension, hyperthermia, and diarrhea) [2]. The typical triad is not present in all cases. However, the toxidrome must be included if several symptoms suggest it and there is a possibility of taking drugs or toxins that have serotonergic effects. Because of the vast diversity and non-specificity of the symptoms, this condition can easily go unrecognized or misdiagnosed, delaying treatment [1].

The diagnosis of SS is made solely on clinical grounds using the Hunter criteria, which is estimated to be 84% sensitive and 97% specific. According to these criteria, SS can be diagnosed by taking a serotonergic agent and the development one of the following findings: spontaneous clonus, inducible clonus or ocular clonus with agitation/diaphoresis, tremor with hyperreflexia, or hypertonic muscle tone with a temperature greater than 100.4°F with ocular or inducible clonus [3,11]. The close differential diagnosis of SS is neuroleptic malignant syndrome (NMS). The clinical manifestations of NMS and SS are remarkably similar, making it difficult to distinguish between the two clinical entities. Patients can fit the criteria for both syndromes. A few other characteristics may assist in distinguishing SS from NMS. NMS is characterized by "lead-pipe" rigidity, while SS is characterized by hyperreflexia and clonus [12]. In some cases, the start and progression of symptoms can be a diagnostic clue. Unlike SS, which has a quick onset and progression of symptoms, NMS usually progresses slowly [12]. Similarly, while SS resolves typically within a few days after ceasing the causative medicines and initiating therapy, NMS typically takes 10-20 days to resolve [2]. The diagnosis of SS should only be confirmed after an infectious, neurological or metabolic pathology has been eliminated.

Treatment initially consists of stopping the offending agent and excellent supportive care [5,12]. Hospitalization with close monitoring of vital signs, renal function, electrolyte, and fluid balance is recommended [5,12,13]. Benzodiazepines are indicated to alleviate agitation and seizures and their consequences, such as hyperthermia [13]. However, resistance to this treatment or symptomatic severity, sedation, and paralysis under intubation is required, as was the case in this patient [5,13]. If the patient has hyperthermia, starting with active external cooling is essential [5,12-13]. Antipyretics are ineffective since the temperature rise is muscular [5]. Because hemodynamic instability can quickly develop into hypotension and shock, arterial hypertension should be treated with short-acting drugs. Finally, cyproheptadine, a serotonin antagonist, has been suggested for the treatment of SS if supportive treatment and benzodiazepines fail to improve agitation and correct vital signs [12,13]. The treatment regimen is, to begin with, a loading dose of 12 mg orally or crushed via the nasogastric tube followed by 2 mg every two hours until the patient is clinically stabilized [2-5]. In any case, restraint by physical restraint should be avoided, as this increases isometric contraction of the muscles, leading to an increase in hyperthermia and lactic acidosis [2].

SS has a fairly good prognosis, and most illnesses are resolved within 24 hours [5]. However, symptoms last longer than 24 hours in some cases, necessitating more stringent treatment [2].

## Conclusions

SS is a potentially life-threatening adverse drug reaction resulting from ingesting substances with serotonergic effects, including psychostimulants such as cocaine. Our case was unique as in our patient; the use of cocaine and fentanyl precipitated SS. It implicates that SS should be suspected, especially in drug abusers treated with psychotropic agents. The clinical presentation is variable and often non-specific, making diagnosis difficult, mainly because it is unfamiliar to practitioners. Training healthcare professionals to recognize the manifestations of SS is undoubtedly a challenge. However, it is imperative to intervene quickly and early in patients to avoid fatal outcomes.
